# Artificial Intelligence–driven Detection, Mapping, and Personalized Therapy for Atrial Fibrillation

**DOI:** 10.19102/icrm.2026.17011

**Published:** 2026-01-15

**Authors:** Daniel Joseph Gonzalez, Samhith Kambampati, Erick Godinez, Ishan Paranjpe, Kushal Chatterjee, Rahul Devathu, Aaryamaan Verma, Emma Sun, Connie Ma, Muhammad Fazal, Tina Baykaner

**Affiliations:** 1 Department of Medicine, Stanford University School of Medicine, Stanford, CA, USA; 2 Department of Medicine, University of Nevada-Reno School of Medicine, Reno, NV, USA; 3 Department of Medicine, The University of British Columbia, Vancouver, Canada

**Keywords:** Artificial intelligence, atrial fibrillation, catheter ablation

## Abstract

Atrial fibrillation (AF), the most common arrhythmia worldwide, affects approximately 59 million people globally. It poses a significant health burden by increasing morbidity and mortality. Artificial intelligence (AI) is emerging as a potentially transformative technology across the AF care continuum. This review synthesizes current evidence and critically evaluates AI applications in AF management, including innovations in detection and screening using electrocardiography and wearables; advanced mapping techniques using signal processing and computational modeling to guide catheter ablation; machine learning-based prediction of treatment outcomes; and personalization of long-term therapy, such as anticoagulation. Key studies and trials illustrating AI’s capabilities in improving diagnostic yield, refining ablation targets, and enhancing prognostic accuracy are analyzed. The potential for AI to facilitate integrated care pathways, such as the “AF Better Care” approach, is considered, balancing innovation against clinical practicality, rigorous validation, and workflow integration. While AI shows considerable potential to augment precision in AF management, significant challenges concerning data generalizability, model interpretability, clinical utility validation, and equitable implementation remain. Optimal integration requires careful alignment with clinical expertise and a focus on patient-centric outcomes. Addressing these challenges through collaborative efforts among clinicians, researchers, and technology developers will be essential to fully realize AI’s promise in improving AF care. Future research should prioritize robust validation, transparent methodologies, and practical implementation strategies to ensure that AI effectively enhances patient outcomes.

## Introduction

Atrial fibrillation (AF) is the most prevalent sustained cardiac arrhythmia, affecting an estimated 59 million individuals worldwide in 2019, a figure projected to rise substantially with population aging and improved detection.^1^ AF significantly increases the risk of ischemic stroke, heart failure, cognitive impairment, and all-cause mortality, while diminishing the quality of life.^2^ Effective management thus requires a comprehensive, patient-centered strategy addressing disease screening, thromboembolism prevention, and symptom management, along with the optimization of cardiovascular risk factors and comorbidities. Guidelines advocate for structured, integrated care models such as the AF Better Care (ABC) pathway.^2^ However, translating guidelines into optimal real-world outcomes is challenged by AF’s complexity, including its often paroxysmal and asymptomatic nature, the heterogeneity in underlying mechanisms and treatment responses, and challenges in integrating risk factor modification.^3^

Timely diagnosis and early intervention are critical in the management of AF. The Early Treatment of Atrial Fibrillation for Stroke Prevention Trial (EAST-AFNET 4) demonstrated that initiating rhythm-control therapy within 1 year of AF diagnosis significantly improves cardiovascular outcomes compared to delayed intervention or minimal rate-control strategies.^4^ Ongoing research continues to investigate the optimal initial management strategy for AF. Recent trials, such as the Early Aggressive Invasive Intervention for Atrial Fibrillation (EARLY-AF) trial, have shown that first-line catheter ablation in treatment-naive patients may be safer and more effective compared to initial anti-arrhythmic drug (AAD) therapy.^5^ Similarly, the Cryoballoon Catheter Ablation in an Anti-arrhythmic Drug Naive Paroxysmal Atrial Fibrillation (STOP AF First) trial found that cryoballoon ablation as an initial therapy was superior to AAD therapy for preventing atrial arrhythmia recurrence in patients with paroxysmal AF.^6^ Despite these advancements, optimizing care remains challenging due to diagnostic difficulties and incomplete understanding of AF pathophysiology, particularly in persistent AF, where complex interactions among triggers, drivers, and atrial substrates occur.^7,8^ These complexities contribute to the variable responses observed with AADs and catheter ablation therapies across the entire AF spectrum.^3^

Artificial intelligence (AI), including machine learning (ML) and deep learning, offers powerful computational tools to address these clinical challenges.^9–11^ By analyzing complex patterns within high-dimensional data from sources such as electrocardiograms (ECGs), wearable devices, medical imaging, and electronic health records (EHRs), AI algorithms can enhance diagnostic accuracy, tailor individualized treatment strategies, predict clinical outcomes, and optimize patient monitoring.^9,12^ However, the clinical integration of these advanced computational tools is not without its challenges. A critical, long-standing argument centers on the need for AI to be explainable; the so-called “black box” problem, where the reasoning behind a model’s output is unclear, can be a significant barrier to clinical trust and adoption.^13^ This review critically appraises current and emerging AI applications across the AF management spectrum, from initial detection and risk stratification to treatment guidance and long-term monitoring. We evaluate the supporting evidence, focusing on innovations poised to enhance diagnostic capabilities, personalize therapeutic strategies, and ultimately improve patient outcomes within the electrophysiology community. The foundational step in managing AF effectively is accurate and timely detection, a domain where AI is demonstrating significant potential to overcome the limitations of traditional screening methods.

## Enhancing atrial fibrillation detection and screening with artificial intelligence

Early and accurate detection of AF is crucial for the timely initiation of guideline-directed therapies and stroke prevention.^2^ Traditional detection methods, such as opportunistic ECG screening, have limited sensitivity for intermittent AF episodes, while prolonged monitoring approaches are constrained by cost and scalability challenges.^3^ AI is enabling novel detection strategies by enhancing clinical ECG interpretation and automating analysis of data from wearables.

### Artificial intelligence–based electrocardiogram analysis: detecting undiagnosed or future atrial fibrillation from sinus rhythm electrocardiograms

A notable advancement leverages deep learning, specifically convolutional neural networks (CNNs), to identify predisposition to AF from standard 12-lead ECGs recorded during sinus rhythm. CNNs are particularly well suited for this task due to their ability to automatically learn hierarchical patterns and subtle morphological features within the ECG signal that may escape human interpretation and traditional signal processing tools. The underlying premise is that early or established atrial cardiomyopathy, a common substrate for AF, imparts subtle but consistent electrocardiographic signatures during sinus rhythm that these complex algorithms can detect.^14^ Foundational research by Attia et al. (2019) demonstrated the feasibility and effectiveness of this AI-enhanced ECG analysis, achieving high discriminative performance (area under the curve [AUC] of 0.87 from a single ECG, rising to 0.90 with multiple ECGs) in identifying patients with a documented history of paroxysmal AF based solely on sinus rhythm ECG data.^14^ This pivotal study established the standard 12-lead ECG as a viable tool for opportunistic AF screening. In the near-term, this could translate into automated flags within EHR systems, alerting clinicians to patients who may warrant extended cardiac monitoring based on routine ECGs. In the medium term, we anticipate that this AI-guided approach could be integrated into formal screening guidelines for at-risk populations, fundamentally changing how we proactively identify individuals with undiagnosed AF.

The prospective clinical utility was investigated in a targeted screening trial.^15^ In this study, an AI-based ECG algorithm stratified older adults (aged ≥65 years) with stroke risk factors, after which subsequent continuous monitoring identified AF significantly more frequently in individuals classified as high risk by AI (7.6%) compared to those classified as low risk (1.6%; *P* < .001). This AI-guided strategy screening approach improved AF detection rates compared to usual care, demonstrating its potential to optimize resource allocation for rhythm monitoring. Moreover, the use of AI-driven analysis has recently expanded to single-lead ECG recordings. Hygrell et al. (2023) reported a reasonable predictive accuracy (AUC, 0.80) for identifying paroxysmal AF from single-lead sinus rhythm ECGs in an age-diverse population.^16^ However, performance varied considerably among more homogenous age subgroups (AUC, 0.62), highlighting how population characteristics can influence the generalizability of predictive models.

### Wearable sensors and artificial intelligence algorithms: scaling atrial fibrillation monitoring

Consumer smartwatches and fitness trackers, equipped with photoplethysmography (PPG) sensors, are enabling large-scale, long-term, passive rhythm monitoring and significantly expanding AF screening capabilities.^2^ Early landmark decentralized studies, such as the Apple Heart Study and the Huawei Heart Study, used proprietary irregular pulse–detection algorithms integrated within smartwatches.^17,18^ These studies demonstrated the feasibility of mass screening via wearables, identifying many individuals with previously unknown AF, often with high positive predictive values (PPVs) for algorithm-triggered notifications (PPV, 84% in the Apple Heart Study for ECG patch confirmation following alert; PPV, 92% in the Huawei Heart Study).^17,18^

The evolution of this technology has rapidly moved toward incorporating sophisticated AI and ML directly into the analysis of these PPG signals. For instance, subsequent research from the Huawei Heart Study detailed the development and validation of a specific ML-based model (using eXtreme Gradient Boosting) for the real-time prediction of AF onset from PPG data. This ML model showed good predictive ability, achieving an AUC of 0.94 for AF onset within 4 h when prospectively validated against 72-h Holter ECG monitoring in a high-risk population.^19^ This work illustrates a direct application of ML to PPG signals for proactive AF risk assessment, moving beyond simple irregular pulse detection.

Robust algorithms are emerging to address the inherent challenges of real-world PPG signal quality, which can be degraded by motion artifacts and noise. Ballinger et al. (2018) introduced DeepHeart, a semi-supervised deep learning model (long short-term memory), which analyzed PPG and activity data from wearables to predict a range of cardiovascular risk factors and related conditions.^20^ Their AI model showed a high accuracy in detecting various medical conditions and achieved AUCs of 0.85 (diabetes), 0.74 (high cholesterol), 0.81 (high blood pressure), and 0.83 (sleep apnea).^20^ Following this, Tison et al. (2018) demonstrated a deep neural network for passive AF detection using smartwatch PPG data, achieving a high AUC of 0.97 against physician-interpreted 12-lead ECGs in a controlled cohort of patients undergoing cardioversion.^21^ Crucially, the authors also reported that the AUC was substantially lower at 0.72 in ambulatory patients, highlighting how real-world conditions can impact performance and underscoring the potential for model overfitting.^21^ These studies highlighted the early potential of AI in leveraging consumer wearable data for AF detection and broader health insights. More recently, Vo et al. (2024) proposed an attention-based deep state space model specifically designed to translate noisy PPG signals into corresponding ECG waveforms.^22^ Their AI model, engineered for noise robustness and data efficiency, achieved a high area under the precision–recall curve of 0.99 for AF detection when the translated ECGs were processed by an existing AF detector. Though this remains experimental and requires further clinical validation, it showcases AI’s advancing capabilities in enhancing signal quality and the diagnostic utility of PPG data.^22^ A near-term implication is the potential for backend integration of such technology to reduce the high rate of “inconclusive readings” from current consumer devices, thereby improving their reliability. In the medium term, this could enable certain wearables to function as near-diagnostic tools, providing high-fidelity rhythm analysis that could streamline the path to definitive diagnosis and treatment.

The regulatory landscape for these technologies is also maturing. As of early 2023, several direct-to-consumer wearable smart devices have received clearance for AF detection, including the Apple Watch (Series 6 and later; Apple, Inc., Cupertino, CA, USA), Samsung Galaxy Watch (3 and later; Samsung Group, Seoul, Korea), Withings ScanWatch (Withings, Issy-les-Moulineaux, France), and Fitbit Sense (Fitbit, Inc., San Francisco, CA, USA), alongside ECG-specific devices such as the KardiaMobile (AliveCor, Mountain View, CA, USA). A clinical validation study by Mannhart et al. (2023) assessed five such smart devices. While sensitivities and specificities for automated AF detection were generally comparable when conclusive readings were obtained, the study highlighted that a significant proportion of tracings, ranging from 17%–26% depending on the device, were deemed inconclusive by the devices’ embedded algorithms.^23^ This finding underscores the ongoing need for algorithmic improvements, potentially through more advanced AI, to reduce inconclusive readings and enhance reliability in real-world scenarios, even as a manual review of these inconclusive tracings by cardiologists proved highly accurate.^23^

### Implications for screening strategies

AI-enhanced ECG analysis may enable opportunistic risk stratification within existing clinical workflows, facilitating targeted deployment of diagnostic resources.^15^ Concurrently, AI-driven analysis of wearable PPG sensor data offers unprecedented scalability for long-term population surveillance and even prediction of AF onset.^19,22^ Optimal screening strategies in the future will likely involve synergistic application of these technologies: AI ECG analysis could identify high-risk individuals during routine clinical encounters, who could then be directed toward targeted long-term monitoring using wearables or clinical-grade devices. The Prediction of Undiagnosed Atrial Fibrillation Using a Machine Learning Algorithm (PULsE-AI) trial, for example, used an ML risk-prediction algorithm based on primary care EHRs (not directly on PPG) to identify individuals at high risk of undiagnosed AF. These individuals were then offered diagnostic testing, which included ECG-based KardiaMobile monitoring.^24^ Although the primary ML algorithm in PULsE-AI was not PPG-based, its study design points toward pathways where AI identifies at-risk cohorts for whom wearable monitoring, potentially including AI-enhanced PPG devices, could be beneficial.

Realizing this potential requires overcoming significant implementation hurdles, including rigorous validation of algorithms across diverse populations, development of efficient clinical pathways for diagnostic confirmation and management initiation, careful consideration of potential harms such as overdiagnosis and patient anxiety, and robust demonstration of cost-effectiveness.^2,3^ Key studies employing advanced AI for AF detection and screening are summarized in **Table 1**.

**Table 1:** Selected Studies Employing Advanced AI for AF Detection and Screening

Study	Population/n	AI Input/Method	Key AI Contribution/Insight	Performance/Outcome Metric(s)	Clinical Implication(s)/Limitation(s)
AI-enhanced detection using ECGs					
Attia et al. (2019)^14^	180,922 patients	12-lead ECG (sinus rhythm)/CNN	Identifies a latent electrocardiographic signature of AF in patients during normal sinus rhythm	Single ECG: AUC, 0.87 Multiple ECGs: AUC, 0.90	Implication: Enables opportunistic AF risk screening using routine, existing 12-lead ECGs Limitation: Retrospective, single-center design requires prospective validation for screening utility
Hygrell et al. (2023)^16^	14,831 patients (≥65 years old) from three screening studies	Single-lead ECG (sinus rhythm)/CNN	Extends the AI ECG concept of identifying latent AF signatures from a 12-lead to a single-lead ECG format	For predicting paroxysmal AF in age-diverse cohorts: AUC, 0.80 For predicting paroxysmal AF in age-homogenous cohorts: AUC, 0.62	Implication: Shows potential for simpler, more accessible single-lead ECG-based AF risk screening Limitation: Performance is significantly influenced by the age diversity of the population
AI-powered detection and prediction using wearable sensors					
Guo et al. (2021)^19^ (Huawei Heart Study)	554 individuals with AF (development) + 50 high-risk patients (validation)	PPG signals/XGBoost ML model	Predicts the onset of AF in real time (0–4 h prior) using continuously monitored PPG signals	For AF onset prediction within 4 h: AUC, 0.94	Implication: Offers a proactive, predictive AF risk assessment that moves beyond simple detection Limitations: Validated in a high-risk population; requires continuous PPG monitoring
Ballinger et al. (2018)^20^ (DeepHeart)	14,011 patients (57,675 person-weeks of data from wearable sensor users)	Wearable PPG and activity data/semi-supervised LSTM network	Predicts multiple cardiovascular risk factors and related conditions using passive wearable sensor data	AUCs for predicting various conditions: Diabetes: 0.85 High cholesterol: 0.74 Sleep apnea: 0.83 Hypertension: 0.81	Implication: Demonstrates the potential for wearables in comprehensive cardiovascular risk stratification beyond AF Limitations: Retrospective analysis; condition labels were based on user self-reporting
Tison et al. (2018)^21^	51 patients (cardioversion cohort) + 9750 participants (validation cohort)	Smartwatch PPG and step count/DNN	Validates passive AF detection accuracy of a smartwatch PPG-based DNN against 12-lead ECGs	Cardioversion cohort: AUC, 0.97 Ambulatory patients: AUC, 0.72	Implication: Establishes high technical accuracy of PPG-based AI in controlled settings Limitations: Real-world performance is reduced by noise and motion artifacts; ECG confirmation is often required
Vo et al. (2024)^22^	55 patients from the MIMIC-III database	PPG signals/ADSSM	Translates noisy PPG signals into ECG waveforms to leverage more advanced ECG-based AF detectors	For AF detection when using the translated ECG signals: PR-AUC, 0.99	Implication: May improve the diagnostic utility of PPG data, especially when signals are noisy Limitations: Small sample size; translation accuracy requires broader validation
AI-guided population screening strategies					
Noseworthy et al. (2022)^15^	1003 patients	12-lead ECG (sinus rhythm)/CNN algorithm	Tests the clinical utility of an AI ECG algorithm to guide a targeted AF screening strategy in a prospective trial	AF detection yield: 7.6% in AI-high-risk group vs. 1.6% in AI-low-risk group after continuous monitoring	Implication: Demonstrates that AI-guided screening can enrich the target population and optimize monitoring resources Limitations: Non-randomized design; the primary outcome was AF detection, not clinical events
Hill et al. (2022)^24^ (PULsE-AI)	23,745 primary care participants (≥30 years)	EHR data/ML risk-prediction algorithm	Identifies individuals at high risk of undiagnosed AF using an EHR-based algorithm for targeted diagnostic testing	Among high-risk participants who underwent testing: 9.41% received an AF diagnosis vs. 4.93% in the high-risk control arm	Implication: EHR-based ML can effectively identify high-risk cohorts for targeted AF screening Limitation: Low uptake of diagnostic testing (28.1% of those invited) limited the overall impact

*Abbreviations:* ADSSM, attention-based deep state space model; AF, atrial fibrillation; AI, artificial intelligence; AUC, area under the receiver operating characteristic curve; CNN, convolutional neural network; DNN, deep neural network; ECG, electrocardiogram; EHR, electronic health record; LSTM, long short-term memory; ML, machine learning; PPG, photoplethysmography; PR-AUC, precision–recall area under curve; XGBoost, eXtreme Gradient Boosting.

## Artificial intelligence–enhanced risk stratification for stroke and atrial fibrillation progression

Beyond initial detection, accurate patient risk stratification is paramount for guiding preventative therapies, particularly for thromboembolic complications such as stroke. While established clinical scores such as CHA₂DS₂-VASc provide essential population-level guidance, their poor predictive accuracy at the individual patient level limits personalized decision-making.^25^ AI and ML models offer a pathway to more granular risk assessment by integrating high-dimensional data encompassing clinical variables, genomic data, biomarkers, and quantitative metrics derived from imaging modalities.^26^ ML analyses applied to large-scale EHR data have demonstrated potential to outperform standard risk scores in predicting thromboembolic events, although performance variability and the need for external validation remain key considerations.^10,11^

AI is also poised to play a vital role in navigating the complexities of subclinical AF, often manifesting as atrial high-rate episodes (AHREs) detected by cardiac implantable electronic devices. Device-detected AF creates significant clinical uncertainty regarding the initiation of anticoagulation therapy, as the balance of benefit and harm differs from that in clinically apparent AF.^27^ A meta-analysis of two recent randomized trials (Non-Vitamin K Antagonist Oral Anticoagulants in Patients With Atrial High Rate Episodes [NOAH-AFNET 6] and Apixaban for the Reduction of Thrombo-embolism in Patients with Device-Detected Subclinical Atrial Fibrillation [ARTESIA]) demonstrated that, while direct oral anticoagulants led to a 32% relative reduction in ischemic stroke, this occurred in the context of a low absolute stroke risk (~1.0% per year in the control arms) and at the cost of a 62% relative increase in major bleeding, highlighting persistent clinical equipoise.^27^

Therefore, AI analysis integrating detailed device data with comprehensive clinical factors may enable identification of the specific AHRE patient subset most likely to derive net clinical benefit from oral anticoagulation (OAC).^27^ Moreover, AI-driven patient phenotyping through unsupervised ML methods, such as clustering algorithms applied to detailed clinical and imaging datasets, can uncover distinct subgroups within the broader AF population. This approach has the potential to refine stroke risk stratification for both clinically evident and subclinical AF by elucidating previously unrecognized patient heterogeneity relevant to clinical outcomes.^28^ Near-term, these AI-defined phenotypes may help clinicians identify candidates for more aggressive risk factor modification or earlier intervention. Looking forward 3–5 years, this could evolve into validated, dynamic risk-stratification tools that supersede current static scores, allowing for more precise, personalized anticoagulation and treatment decisions.

Optimizing OAC requires a careful balance between thromboembolic prevention and mitigating bleeding risk.^2^ As previously noted, established clinical risk scores possess limited predictive capacity at the individual level.^25^ ML algorithms integrating broader, longitudinal data from EHRs, potentially including genomic or biomarker data, may offer more precise and dynamic predictions of both ischemic and bleeding events. This could refine OAC decision-making, particularly for patients with intermediate risk scores; inform choices between OAC and left atrial (LA) appendage closure; or guide management in challenging scenarios such as subclinical AF.^25–27^

## Artificial intelligence in atrial fibrillation ablation: enhancing mapping, guidance, and outcome prediction

Catheter ablation, primarily centered on pulmonary vein isolation (PVI), is an established effective therapy for paroxysmal AF. However, its success rates are considerably lower for persistent and long-standing persistent AF, likely attributable to complex extra-pulmonary vein arrhythmogenic mechanisms and progressive atrial substrate remodeling.^2,7,8^ Conventional electroanatomic mapping techniques often struggle to reliably characterize these patient-specific mechanisms or the underlying substrate alterations.^29^ AI can interpret and integrate the complex electrical and structural patterns underlying fibrillatory conduction, enabling mechanism-targeted, personalized approaches to strategies that extend beyond PVI.

### Artificial intelligence–enhanced electrophysiological mapping

AI algorithms are being developed to analyze complex intracardiac electrograms (EGMs) automatically, aiming to identify regions critical for AF initiation or perpetuation. ML techniques, particularly deep learning architectures such as CNNs, can automate the classification of complex EGM features and standardize the interpretation of AF-activation patterns in persistent AF.^30^ However, because these sophisticated models can function as “black boxes,” their clinical acceptance hinges on transparency. To address this directly, explainable AI (XAI) methods are now being used to provide clinicians with clear insights into the features and rationale underlying these classifications, moving beyond a simple prediction to offer a justification for the algorithm’s conclusions.^30^ Additionally, ML approaches analyzing frequency-domain characteristics of EGMs, such as dominant frequency and organization indices derived from Fourier spectra, have shown promising results when validated against high-resolution optical mapping of ex vivo human hearts, potentially surpassing traditional spectral analysis methods in detecting AF drivers.^31^

Emerging AI-driven mapping systems aim to transform EGM pattern recognition directly into actionable ablation targets. For example, AI analyses that measure spatiotemporal dispersion within EGMs can identify areas of highly disorganized conduction, hypothesized to reflect proximity to critical AF drivers.^32^ This approach was prospectively evaluated in the randomized Tailored vs. Anatomical Ablation Strategy for Persistent Atrial Fibrillation (TAILORED-AF) trial, demonstrating that ablation of AI-identified dispersion zones, in addition to standard PVI, significantly improved 12-month freedom from AF compared to PVI alone (89% vs. 67%, *P* < .001) in patients with persistent AF.^33^ The near-term implication for practice is the potential for wider adoption of this specific AI-guided strategy in electrophysiology laboratories for ablation of persistent AF cases, acquiring broader intraprocedural and follow-up data for AI model training, and improving the algorithm’s accuracy for achieving procedural efficiency and acute endpoints. Over the medium term, this success could establish a new standard of care for substrate modification in persistent AF, contingent on long-term data and broader availability of the technology.

### Computational modeling for personalized ablation planning

An alternative, in silico approach involves creating patient-specific computational models of the atria, typically based on high-resolution imaging such as late gadolinium enhancement magnetic resonance imaging (LGE-MRI), which delineates both atrial anatomy and fibrosis distribution. By incorporating detailed mathematical representations of atrial electrophysiology, including conduction velocity, refractoriness, and fibrosis-driven tissue heterogeneity, these models can non-invasively simulate AF dynamics.^34^ The principal objective is to identify patient-specific mechanisms responsible for AF maintenance, particularly re-entrant driver locations linked to fibrotic substrates, thereby facilitating precise, individualized ablation target selection before the clinical procedure. The Optimal Target Identification via Modelling of Arrhythmogenesis (OPTIMA) strategy demonstrated the practical feasibility of using such computational simulations to detect existing as well as emergent AF drivers associated with fibrosis distribution, enabling formulation of a comprehensive, patient-tailored ablation plan in advance.^34^ Although computationally demanding, this strategy holds considerable promise for non-invasive, mechanistically guided ablation planning. Furthermore, integrating simulation-derived functional parameters into ML frameworks has shown potential for predicting AF recurrence post-ablation, underscoring that computational modeling effectively captures clinically relevant aspects of the atrial substrate.^7^

### Artificial intelligence for predicting ablation outcomes

Accurate prediction of catheter ablation success is essential for effective patient selection, expectation management, and tailoring individualized therapeutic strategies.^9,35^ ML models are demonstrating significant potential to improve prognostication by integrating diverse, high-dimensional data sources beyond traditional clinical risk factors. Multimodal AI models, which incorporate various data sources, including intracardiac EGMs collected during ablation, pre-procedural surface ECGs, clinical variables, raw imaging studies, and imaging-derived metrics, consistently outperform simpler clinical scoring systems in predicting AF recurrence post-ablation.^35,36^ For example, Tang et al. (2022) demonstrated that a deep learning fusion model combining intracardiac EGMs, ECGs, and clinical parameters yielded superior accuracy (AUC, 0.86) for predicting 1-year AF recurrence compared to single-modality models or clinical scores alone.^35^ Similarly, Razeghi et al. (2023) reported robust predictive performance (AUC, 0.82) by integrating clinical data with morphological and deep learning–derived features extracted from pre-ablation cardiac computed tomography (CT) images.^36^ Further demonstrating the power of data fusion, Roney et al. (2022) showed that combining patient history and imaging data with metrics from biophysical in silico simulations significantly improved the prediction of long-term AF recurrence (AUC, 0.85) compared to models using clinical and imaging data alone (AUC, 0.66).^37^ Novel imaging biomarkers further contribute to predictive accuracy; Firouznia et al. (2021) illustrated that CT-derived fractal features assessing LA shape and texture complexity substantially improved recurrence prediction (AUC, 0.87 when combined with clinical data) compared to LA volume alone (AUC, 0.59).^38^ Collectively, the near-term implication of these advanced multimodal models is their use as powerful adjunctive tools in shared decision-making, enabling more personalized discussions about the likelihood of ablation success. In the medium term (3–5 years), as these analytics undergo prospective validation and integration into clinical platforms, they could directly guide patient selection for ablation and help tailor procedural strategies to individual substrate characteristics.

Quantitative fibrosis assessment derived from LGE-MRI has proven especially valuable as input data for predictive models. By incorporating fibrosis information into personalized computational models, researchers can functionally assess atrial substrates. Shade et al. (2020) demonstrated that features derived from in silico simulations of AF inducibility based on pre-ablation LGE-MRI were highly predictive of recurrence after PVI for paroxysmal AF (validation AUC, 0.82).^7^ Furthermore, unsupervised ML techniques such as clustering, when applied to clinical and LGE-MRI fibrosis data from the Efficacy of Delayed Enhancement MRI-Guided Ablation vs. Conventional Catheter Ablation of Atrial Fibrillation (DECAAF II) trial, identified distinct phenotypes within persistent AF patients. Specifically, a high-risk phenotype, characterized by older age, higher body mass index, increased LA volume, and greater fibrosis burden, showed significantly elevated recurrence rates post-ablation (51.7% vs. 35.0%), highlighting ML’s capability to reveal clinically relevant patient heterogeneity.^28^ Atrial structural characteristics also influence other outcomes, such as the likelihood of functional mitral regurgitation resolution following successful AF ablation.^39^

Collectively, ML models represent a significant advance in predicting AF ablation outcomes. By integrating complex multimodal data, they offer more accurate, personalized risk stratification, facilitating improved patient selection, enhancing shared decision-making, managing expectations, and potentially paving the way for personalized ablation strategies.^28,35,36^ Widespread clinical adoption requires further validation, particularly in prospective settings, and demonstration of clinical utility beyond predictive accuracy. Selected studies highlighting these AI applications in AF treatment guidance and outcome prediction are presented in **Table 2**.

**Table 2:** Selected Studies on AI for AF Ablation Guidance and Outcome Prediction

Study	Population/n	AI Input/Method	Key AI Contribution/Insight	Performance/Outcome Metric(s)	Clinical Implication(s)/Limitation(s)
Imaging-based and predictive AI applications					
Shade et al. (2020)^7^	32 patients (paroxysmal AF)	LGE-MRI + computational modeling (AF inducibility simulation)/ML classifier	Predicts recurrence risk by integrating mechanistic simulation results with an ML model	In silico simulation features were highly predictive of post-PVI recurrence (validation AUC, 0.82)	Implication: Proof of concept for using functional substrate assessment via simulation for prognosis Limitation: Small, retrospective study focused on paroxysmal AF
Noujaim et al. (2025)^28^ (DECAAF II)	815 patients (persistent AF from DECAAF II trial)	Clinical + LGE-MRI fibrosis data/unsupervised ML clustering (GBM)	Identifies distinct patient phenotypes within persistent AF based on clinical and imaging data	High-risk phenotypes (older, high BMI/LA vol/fibrosis) had significantly higher recurrence rates post-ablation of 51.7% vs. 35.0% (AUC, 0.87)	Implication: Unsupervised ML can reveal clinically relevant patient heterogeneity that is linked to prognosis Limitation: Post-hoc analysis of RCT data
Boyle et al. (2019)^34^ (OPTIMA)	10 patients (persistent AF)	LGE-MRI + computational modeling	Predicts personalized ablation targets in silico based on patient-specific fibrosis substrate	Feasibility of pre-procedural computational target identification was demonstrated	Implication: Potential for non-invasive, mechanism-based ablation planning Limitations: Small feasibility study; computationally intensive; clinical outcome impact not yet proven in an RCT
Razeghi et al. (2023)^36^	321 patients (mixed AF)	Cardiac CT (morphological + deep features) + clinical data/ML fusion framework	Predicts recurrence using a multimodal ML model that fuses imaging and clinical features	Fusion model (AUC, 0.82) was superior to a model using clinical data alone (AUC, 0.63)	Implication: Demonstrates the value of deep learning features from standard CT scans for prognostication Limitations: Retrospective design; requires external validation
Roney et al. (2022)^37^	100 patients (mixed AF)	MRI, clinical data + biophysical simulation metrics/ML classifier (SVM)	Fuses clinical data with patient-specific simulation “stress tests” to improve prediction of long-term recurrence	Combined model (AUC, 0.85) significantly outperformed clinical/imaging model alone (AUC, 0.66)	Implication: Demonstrates that integrating functional data from simulations can substantially boost predictive accuracy Limitations: Requires significant computational resources; prospective validation needed
Firouznia et al. (2021)^38^	203 patients (mixed AF)	Cardiac CT (fractal features) + clinical data/random forest classifier	Predicts recurrence using novel CT-derived fractal features of LA shape and texture	Combination model achieved superior prediction (AUC, 0.87) compared to LA volume alone (AUC, 0.59)	Implication: Highlights predictive value of quantitative LA morphology beyond simple volumetric measurement Limitations: Retrospective analysis; fractal feature extraction can be complex
Bautista et al. (2023)^39^	50 patients (AF with moderate-severe functional MR)	Post-ablation 3D voltage mapping/analysis of low-voltage zones (scars)	Predicts a non-arrhythmic outcome (MR resolution) based on atrial substrate characteristics	Presence of a posterior bottom LA scar was an independent predictor of refractory MR after AF ablation	Implication: Suggests that atrial substrate remodeling impacts mechanical function recovery, not just rhythm outcome Limitation: Small, retrospective study
Intraprocedural signal analysis AI applications					
Reddy et al. (2024)^8^ (FLOW-AF)	85 patients (redo non-paroxysmal AF)	Intracardiac EGM (electrographic flow)/EGF mapping algorithm	Guides source ablation by targeting AI-reconstructed activation sources (EGF) in a prospective RCT	EGF-guided ablation + PVI was superior to PVI alone for 12-month AF-free survival (68% vs. 17%)	Implication: RCT evidence supports AI-guided source ablation benefit in a challenging redo persistent AF population Limitations: Smaller sample size; specific algorithm; focus on redo procedures
Alhusseini et al. (2020)^30^	35 patients (persistent AF)	Intracardiac EGM/CNN with explainable AI (XAI)	Classifies EGM patterns objectively using deep learning and provides explanations for the AI’s logic	High accuracy (95%) for classifying rotational vs. non-rotational activity	Implication: Potential for automated, objective EGM analysis with increased clinical trust via XAI Limitations: Proof-of-concept design; small sample size; clinical utility not yet established
Zolotarev et al. (2020)^31^	11 explanted human atria	Intracardiac EGM (Fourier spectra)/ML (various classifiers)	Identifies AF driver regions by analyzing EGM frequency features, validated against optical mapping	Higher-density catheter datasets had higher F1-score (0.81) vs. lower-density catheter dataset (F1-score, 0.66)	Implication: Supports using ML for objective driver identification from EGM frequency content Limitations: Ex vivo study; performance is dependent on mapping density
Bars et al. (2023)^32^	71 patients (persistent AF)	Intracardiac EGM/AI software for spatiotemporal dispersion mapping	Characterizes spatiotemporal dispersion patterns and compares them between induced and spontaneous AF	Dispersion distribution was similar, but global dispersion was higher in spontaneous LA-AF (15% vs. 10%)	Implication: Foundational study for TAILORED-AF, showing that dispersion patterns differ by AF induction method Limitations: Observational; characterizes patterns; does not directly assess ablation outcome
Deisenhofer et al. (2024)^33^ (TAILORED-AF)	374 randomized patients (persistent AF)	Intracardiac EGM (spatiotemporal dispersion)/AI software (Volta VX1)	Guides substrate ablation by targeting AI-identified EGM dispersion markers in a prospective RCT	AI-guided ablation + PVI was superior to PVI alone for 12-month AF-free survival (89% vs. 67%)	Implication: RCT evidence supports AI-guided substrate ablation benefit in persistent AF Limitations: Interim analysis of a trial using specific AI software; long-term outcomes awaited
Tang et al. (2022)^35^	156 patients (mixed AF)	Intracardiac EGM + 12-lead ECG + clinical data/CNN + fusion ML	Predicts 1-year ablation recurrence using a multimodal fusion of electrophysiologic and clinical data	Superior prediction (AUC, 0.86) vs. single-modality models or clinical scores alone: EGM signals (AUC, 0.73) APPLE scores (AUC, 0.64) CHA_2_DS_2_-VASc scores (AUC, 0.65)	Implication: Demonstrates the value of ML fusion integrating diverse data sources for prognostication Limitations: Retrospective design; requires external validation

*Abbreviations:* AF, atrial fibrillation; AI, artificial intelligence; AUC, area under the receiver operating characteristic curve; BMI, body mass index; CNN, convolutional neural network; CT, computed tomography; ECG, electrocardiogram; EGF, electrographic flow; EGM, electrogram; GBM, gradient boosting method; LA, left atrium; LGE, late gadolinium enhancement; ML, machine learning; MR, mitral regurgitation; MRI, magnetic resonance imaging; PVI, pulmonary vein isolation; RCT, randomized controlled trial; XAI, explainable artificial intelligence.

## Artificial intelligence in therapy monitoring and personalization

Effective long-term AF management necessitates continuous surveillance, dynamic risk factor modification, and therapy adjustments, ideally within an integrated care framework.^2,3^ AI presents opportunities to enhance the efficiency, personalization, and proactivity of this chronic care phase.^9,11^

### Intelligent monitoring and management adjustment

The substantial volume of data generated by long-term monitoring devices, such as implantable loop recorders (ILRs), pacemakers, and wearables, is well suited for AI-driven analysis.^9^ AI algorithms can automate AF detection and burden quantification more accurately from these continuous data streams, aiding in the objective assessment of treatment efficacy (eg, response to AAD or ablation).^12^

ILRs often generate false-positive AF detections, frequently triggered by events such as premature atrial contractions. AI-based filters, however, can significantly improve the diagnostic accuracy of ILRs. Mittal et al. (2021) demonstrated that one such AI filter improved the PPV of ILR-detected AF episodes from 53.9% to 74.5%, while only minimally affecting the detection of true AF.^40^ Building on this, AI algorithms integrated into ILR systems can substantially decrease the transmission of these false-positive alerts and episodes. Middeldorp et al. (2025) showed that these AI algorithms reduced the alert burden by approximately 20% and the episode burden by around 40%, which can help alleviate clinic workload.^41^

Beyond improving current detection accuracy, predictive analytics applied to longitudinal rhythm and activity data might forecast clinically relevant events, such as progression from paroxysmal to persistent AF or impending heart failure decompensation, potentially enabling preemptive interventions.^11^ While still largely developmental, AI could potentially identify patient-specific AF triggers from wearable sensor data (combining rhythm, activity, sleep, etc.) or assist in personalizing AAD selection and dosing based on predicted efficacy and side effect profiles derived from patient phenotypes.^3,9^ Given the documented discrepancies between guideline recommendations and real-world AAD prescribing practices, AI-based clinical decision support tools could offer valuable assistance, although robust validation is required.^3^

### Enhancing patient engagement and integrated care

AI technologies can also bolster patient engagement and facilitate integrated care models. Natural language processing techniques can analyze patient communications (eg, portal messages) to identify concerns or unreported symptoms.^9^ Beyond direct patient communications, natural language processing can also extract crucial information from narrative EHR text, such as clinical notes and reports, to more accurately define risk factors and improve the prediction of future AF incidence.^42^ Furthermore, conversational AI, in the form of large language models, is being explored for its potential to deliver personalized education on AF. Studies evaluating these large language models have found that they can provide accurate responses to many patient-centered questions regarding AF.^43,44^ For instance, ChatGPT-4 demonstrated high accuracy for patient-level queries^44^ and showed improvement in its responses over time.^43^ However, the reliability of these models for more complex, provider-level questions is currently lower, and they may not always reflect the most recent guidelines or nuances in clinical management. The readability of large language model (LLM)-generated content also varies, sometimes requiring a college-level understanding, which is an important consideration for patient education.^43,44^ AI-enhanced digital dashboards integrated with EHRs can provide clinicians with real-time, synthesized views of patient status, incorporating data from multiple sources (EHRs devices, patient-reported outcomes) to support timely and informed interventions. LLMs may also streamline clinician interaction with complex EHR data, facilitating efficient information retrieval and summarization.^9^ Collectively, these applications aim to render chronic AF management more proactive, data-informed, personalized, and collaborative, aligning strongly with the principles of integrated care pathways like the ABC framework.^2^

## Limitations and future directions

Despite rapid advancements and considerable promise, significant limitations must be addressed for the effective and equitable clinical translation of AI in AF management.^9–12^ The performance of AI models is heavily dependent on the quality, size, and representativeness of the training data. Algorithms validated on data from specific populations or health care systems may lack generalizability when applied to new, diverse settings. Robust external validation across multicenter, heterogeneous datasets is therefore critical but remains frequently lacking in the published literature.^28,35^ Data fragmentation across institutions and incompatible data formats further hinder the development and validation of broadly applicable AI tools.^9^

A fundamental barrier to widespread clinical integration is the inherent complexity of many advanced AI models, particularly deep learning algorithms, which often results in a “black box” phenomenon where the reasoning behind a prediction is not transparent. This lack of interpretability, as highlighted in recent clinical commentary, can erode clinical trust, hinder adoption, and make it challenging to identify and mitigate potential biases encoded within the algorithm.^13,30^ The development and application of XAI techniques are therefore vital to foster understanding and confidence among clinicians. Practical integration into existing clinical workflows represents another substantial hurdle; AI tools require intuitive user interfaces and seamless integration with EHR systems to avoid disrupting established care processes and adding to the clinician burden.^9^ The resource implications and workflow adjustments required for complex AI applications, such as advanced intraprocedural mapping systems or computationally intensive pre-procedural planning, must be carefully balanced against their demonstrated clinical benefits.^8,29,33,34^

Crucially, the ultimate measure of success for any AI application in medicine is the demonstration of improved patient-important clinical outcomes, not merely technical accuracy or predictive performance. Rigorous prospective studies, ideally randomized controlled trials (RCTs), are paramount to establish the clinical utility and real-world effectiveness of AI-driven strategies.^3,4,8,15,33^ Furthermore, comprehensive cost-effectiveness analyses are necessary, particularly for resource-intensive AI solutions, to justify their adoption within health care systems.^7,36^ Ensuring equitable access to AI-enabled care and actively mitigating algorithmic bias, which could potentially exacerbate existing health disparities, are critical ethical considerations that must be proactively addressed.^11^ Agile and appropriate regulatory frameworks are also required to oversee the development, validation, and implementation of clinical AI tools.^9^

Beyond technical hurdles, the deployment of AI in clinical practice is governed by profound ethical considerations that are paramount for responsible innovation.^45^ A primary concern is algorithmic bias, where models trained on non-representative data can perpetuate or even worsen health disparities for underrepresented racial, ethnic, or socioeconomic groups. Equally important are the principles of data privacy and security, which mandate robust governance to protect sensitive patient information from unauthorized access or misuse, in line with regulations such as the General Data Protection Regulation (GDPR). Furthermore, the opacity of many AI models raises complex questions of accountability and liability; determining responsibility when an AI-driven recommendation contributes to an adverse outcome is a critical challenge for clinicians, institutions, and developers alike. Achieving true digital inclusion also requires moving beyond simple access to address a range of interwoven factors. This involves creating a comprehensive ethical framework that systematically addresses not only social but also the crucial digital and commercial determinants of health (such as digital literacy and the market-driven nature of consumer wearables) to ensure that the benefits of AI in AF care do not create a new digital divide.^46^ Proactively establishing ethical frameworks to address these issues of bias, privacy, accountability, and equity is a non-negotiable prerequisite for building AI systems that are not only effective but fundamentally trustworthy.^45^

Future priorities should focus on large-scale, prospective validation studies emphasizing clinical utility and patient outcomes across diverse, representative populations. Standardization of methodologies for AI development, validation, and reporting (akin to Consolidated Standards of Reporting Trials [CONSORT] guidelines for RCTs) would enhance transparency and comparability across studies. Continued advancements in XAI are needed to improve model transparency and trustworthiness. Ultimately, successful and responsible integration of AI into AF care requires sustained collaboration among clinicians, data scientists, engineers, regulators, health care administrators, and patients. The synergy between data-driven ML approaches and mechanistic computational modeling represents a particularly promising frontier for developing more robust and interpretable AI solutions.^7,26,34^

## Conclusions

AI is rapidly evolving from a conceptual promise into a suite of tangible tools poised to significantly influence the diagnosis and management of AF across its entire spectrum. AI-augmented analysis of ECG and wearable sensor data is enhancing the efficiency and reach of AF detection and screening initiatives. Within the electrophysiology laboratory, AI-driven mapping techniques provide patient-specific insights to guide more targeted and potentially more effective catheter ablation strategies, with emerging RCT evidence supporting improved rhythm outcomes for specific patient subgroups. ML models demonstrate superior accuracy over traditional risk scores in predicting treatment outcomes, particularly for ablation, by adeptly integrating complex, multimodal patient data. Furthermore, AI presents compelling opportunities to optimize long-term AF care through more personalized anticoagulation strategies, intelligent remote monitoring, and potentially tailored pharmacotherapy selection and adjustment.

However, translating this considerable potential into widespread, effective clinical practice requires navigating significant challenges. Rigorous prospective validation demonstrating improved, patient-centric outcomes in diverse real-world populations is essential. Addressing critical issues of model interpretability and bias, ensuring seamless and efficient workflow integration, demonstrating cost-effectiveness, and establishing robust regulatory and ethical frameworks are non-negotiable prerequisites for responsible adoption. The role of AI must be carefully cultivated as a powerful adjunct to, rather than a replacement for, clinical judgment, shared decision-making, and established principles of integrated care. Continued interdisciplinary collaboration and focused research addressing these key challenges are paramount to fully harness AI’s transformative potential for improving the lives of patients with AF worldwide.
